# Efficacy and safety of enoxaparin as deep vein thrombosis prophylaxis in critically ill patients

**DOI:** 10.1186/cc11027

**Published:** 2012-03-20

**Authors:** R Al-Hubail, N Hassan

**Affiliations:** 1 King Fahad Specialist Hospital, Dammam, Saudi Arabia

## Introduction

Critically ill patients are at high risk of developing deep vein thrombosis (DVT). DVT cannot be detected in most cases, leading to fatal embolic manifestations [1]. The goal of this study was to review the occurrence of DVT in patients receiving enoxaparin during their length of stay in the ICU (ICU LOS). In addition we review the occurrence of major bleeding and thrombocytopenia secondary to enoxaparin.

## Methods

This was a retrospective database analysis including medical and surgical patients admitted to a tertiary hospital (King Fahad Specialist Hospital Dammam) critical care department from 1 January to 31 December 2010, aged 17 to 70 years, excluding patients with: platelets <50,000/l; evidence of active bleeding; new ischemic or haemorrhagic stroke; spinal or epidural catheter who were already on anticoagulant when admitted to the ICU and who were previously diagnosed with DVT or with pulmonary embolism (PE); DNR (do not resuscitate). The APACHE II score, predicted mortality and ICU LOS were calculated for included patients in the study. The hospital electronic system and critical care database were reviewed with the physician order sheet according to the ICU protocol for DVT prophylaxis (enoxaparin 40 mg subcutaneously once daily).

## Results

Five hundred and ninety-seven patients were investigated, from which 22 (3.5%) fulfilled exclusion criteria, 220 (36%) were on a sequential decompression device (SD), and 26 (4%) were not on DVT prophylaxis (protocol violation). This gave a study population of 329 (55%) cases that were on enoxaparin thromboprophylaxis. In this population there were no recorded cases of DVT and two cases (0.6%) of PE. Major bleeding was recorded in seven cases (2.1%), platelets <50,000/l in eight cases (2.4%), and Hb level <1.5 g/dl from baseline without bleeding in 47 cases (14.2%). See Table 1.

**Table 1:** 

Type of case	ICU LOS (days)	APACHE II	Predicted mortality (%)
Total cases on enoxaparine	5.29 ± 7.3	16.7 ± 10.5	28.4 ± 23.7
Low platelets cases	11.75 ± 9.7	23 ± 2.3	48.75 ± 11.18
Major bleeding cases	13.5 ± 13.1	22.4 ± 17.4	30 ± 17.5

## Conclusion

Using the hospital and critical care databases, we observed that the critically ill patients receiving enoxaparin as thromboprophylaxis did not experience DVT, and two (0.6%) had PE during their ICU stay. However, thrombocytopenia and major bleeding were recorded at very low frequencies (2.5%).
